# Silicone Intubation Indications in External Dacryocystorhinostomy

**Published:** 2014

**Authors:** Ibrahim Bulent Buttanri, Didem Serin

**Affiliations:** Haydarpasa Numune Education and Research Hospital, İstanbul, Turkey

**Keywords:** Silicone Intubation, External Dacryocystorhinostomy, Surgical Technique

External dacryocystorhinostomy (DCR) is the gold standard surgical technique for the treatment of nasolacrimal duct obstruction (1). Since its introduction by Gibbs in 1967, intubation with silicone tubes has been widely used in lacrimal duct surgery (2). However, effect of silicone intubation on patency of lacrimal system during external DCR in cases of primary nasolacrimal duct obstruction (NLDO), canalicular obstruction or in revision surgeries is still controversial.

We do not perform, in our facility, silicone intubation in primary NLDO except in cases with intraoperative complications such as iatrogenic canalicular trauma or flap rupture. There are only a few reports on beneficial effect of silicone intubation on the success rates. On the other hand, more reports demonstrated no significant difference between the success rates of routine external DCR irrespective of silicone intubation (3). Freng et al. performed a meta-analysis based on a literature search and they did not find any benefit for silicone intubation in primary DCR (4).

Silicone tube may cause some complications such as canalicular or punctal slitting, and granuloma formation in the nose and lacrimal fossa (5,6). Kim et al. concluded that prolonged silicone intubation is related with Pseudomonas aeruginosa infections and final surgical failure (7).

In our institution, intubation is performed in revision surgeries and in cases with canalicular problems. We believe that a silicone tube only acts, in a way, as a guide for the canalicular epithelium to progress farther when there is its discontinuity, without adhering to other tissues. Moreover, it ensures patency during the healing process. We evaluated the outcome of silicone intubation in external DCR patients with distal canalicular or common canalicular obstructions and we concluded that silicone intubation is indicated in patients with canalicular problems (8). Boboridis et al. reported a success rate of 92% after external DCR with membranectomy and this ratio is comparable with the outcome of routine DCR procedures (9).

Silicone intubation has some functional and mechanical effects on epiphora. Silicone tube itself may have a mechanical obstructive effect on the canalicular system. However, silicone intubation enhances lacrimal pump function by supporting punctal position and apposition during blinking. Silicone intubation also increases lacrimal tear drainage via increasing capillarity. Kim et al. (10) reported that silicone intubation decreases epiphora in patients after anatomically successful but functionally unsuccessful DCR surgeries.

Silicone intubation increases cost and prolongs postoperative follow-up periods of the patients. Based on current medical evidences and our experience, silicone intubation is mainly indicated in DCR surgery in selected cases of distal or common canalicular obstructions and routine intubation can be avoided in cases with primary NLDO.
